# Predictive value of urethral sphincter complex volume for persistent high voiding pressure in female urethral diverticulum patients post-diverticulectomy

**DOI:** 10.1007/s00345-025-05719-w

**Published:** 2025-06-05

**Authors:** Jen-Chieh Chen, Po-Hsun Pan, Chih-Chieh Lin, Alex T. L. Lin, Eric Yi-Hsiu Huang, Yu-Hua Fan

**Affiliations:** 1 Department of Urology, Taipei Veterans General Hospital, No. 201-7, Sec. 2, Shipai Rd., Beitou Dist, Taipei, Taiwan (ROC); 2 Department of Urology, National Yang Ming Chiao Tung University Hospital, Yilan, Taiwan; 3 Department of Urology, College of Medicine and Shu-Tien Urological Research Center, National Yang Ming Chiao Tung University, Taipei, Taiwan

**Keywords:** Urethral diverticulum, Diverticulectomy, Urethral diverticulum volume, Urethral sphincter complex volume, High-tone nonrelaxing sphincter

## Abstract

**Purpose:**

This study evaluated the relationship between urethral diverticulum volume (UDv), urethral sphincter complex volume (USCv), clinical and urodynamic characteristics, and surgical outcomes in women with UD.

**Methods:**

A retrospective study was conducted on 53 female patients who underwent diverticulectomy for UD. They were divided into two groups: proximal/middle UD (pmUD, *n* = 43) and distal UD (dUD, *n* = 10). Preoperative assessments included videourodynamic study (VUDS) and magnetic resonance imaging, with a follow-up VUDS 1 month post-surgery.

**Results:**

Compared to the dUD group, pmUD patients were less likely to present with a vaginal lump (37.2% vs. 80.0%, *p* = 0.031) but had significantly larger USCv (9.96 ± 5.91 cm^3^ vs. 5.23 ± 1.19 cm^3^, *p* < 0.001) and UDv (6.27 ± 7.40 cm^3^ vs. 0.84 ± 0.81 cm^3^, *p* < 0.001). The pmUD patients had longer surgeries and higher recurrence rates (32.6% vs. 0%, *p* = 0.018). In the pmUD group, 24 patients had bladder outlet obstruction (BOO) and 25 exhibited high-pressure voiding (> 20cmH_2_O). No significant association was found between UDv or USCv and recurrence, unresolved BOO, or high-pressure voiding status. However, a USCv cutoff of ≥ 8.2 cm^3^ was identified, predicting unresolved high-pressure voiding status post-surgery (*p* = 0.023). Additionally, 9.3% pmUD patients developed de novo stress urinary incontinence, correlated with larger UDv (11.5 ± 14.3 cm^3^ vs. 5.9 ± 6.4 cm^3^, *p* = 0.02).

**Conclusion:**

A USCv cutoff of ≥ 8.2 cm^3^ was identified as a predictor for unresolved high-pressure voiding after diverticulectomy.

**Supplementary Information:**

The online version contains supplementary material available at 10.1007/s00345-025-05719-w.

## Introduction

Urethral diverticula (UD) are defined as localized outpouchings of the urethral mucosa into surrounding nonurothelial tissues [1]. The incidence of UD in adult females is estimated at fewer than 20 cases per 1,000,000 (< 0.02%) annually [1], with occurrences in men or children being extremely rare. UD symptoms are varied and often difficult to detect [2]. It may be asymptomatic or found incidentally during physical examination or imaging. The classic symptom triad, known as the “3 D’s,” includes postvoid dribbling (4–31%), dysuria (9–55%), and dyspareunia (6–24%) [3]. Other common symptoms include painful vaginal masses associated with postmicturition incontinence, urinary incontinence (35–39%), frequent urinary tract infections (9–61%), and the presence of stones or tumors [4].

A comprehensive evaluation and diagnosis of UD require a detailed medical history, physical examination, endoscopic evaluation of the bladder and urethra, and specific radiologic imaging [5]. T2-weighted postvoid magnetic resonance imaging (MRI) is the most accurate diagnostic method, with nearly 100% sensitivity and specificity. Additionally, videourodynamic study (VUDS) aids diagnosis in 62–95% of cases, providing important information on voiding dysfunction or stress urinary incontinence, which occurs in up to 49% of cases [5].

The primary treatment for UD is surgical excision. Transvaginal excision, followed by periurethral tissue reconstruction, has shown a success rate of 84–98%. However, studies with longer follow-up indicate that reoperation may be required in up to 13% of cases [6].

The exact cause of acquired UD in women is often unclear, though it has been linked to factors such as blockage of periurethral glands, prior urethral surgery or dilation, and traumatic vaginal delivery [7]. Mukhtar et al. suggested that elevated pressure in the proximal urethra during voiding, due to a high-tone nonrelaxing sphincter (HTNRS), may contribute to UD formation [8]. HTNRS is diagnosed by detecting a high pulsatile maximal urethral closure pressure (MUCP) in the absence of other obstructive causes [9]. Wiseman et al. [10] hypothesized that a nonrelaxing sphincter leads to sphincter hypertrophy, increasing sphincter volume. They demonstrated that urethral sphincter complex volume (USCv), measured via ultrasonography, correlates with MUCP, with higher USCv linked to elevated MUCP [11]. Furthermore, USCv can be measured using MRI, reducing operator dependence [12, 13]. Thus, a raised USCv, in the absence of other causes, can be used as a marker for diagnosing HTNRS.

Since an elevated USCv may suggest a different cause for UD, it could affect both its presentation and treatment outcomes. This study aimed to assess the relationship between USCv, clinical symptoms, urodynamic characteristics, and treatment outcomes in women with UD.

## Materials and methods

A retrospective review of case notes was performed for all women who underwent diverticulectomy for symptomatic UD at a single center from January 2003 to December 2023. The study received approval from the hospital’s Ethics Committee (Institutional Review Board (IRB) number: TPEVGH-IRB 2023-03-018AC). Detailed personal and medical histories were collected through patient interviews. Lower urinary tract symptoms were evaluated using the International Prostate Symptom Score, which includes seven questions addressing voiding symptoms (incomplete emptying, intermittency, weak stream, and straining) and storage symptoms (frequency, urgency, and nocturia) [14]. All patients underwent preoperative assessments, including MRI and VUDS. Postoperative evaluations were conducted with VUDS 1 month after diverticulectomy to assess UD recurrence and changes in voiding dysfunction.

**Fig. 1:**
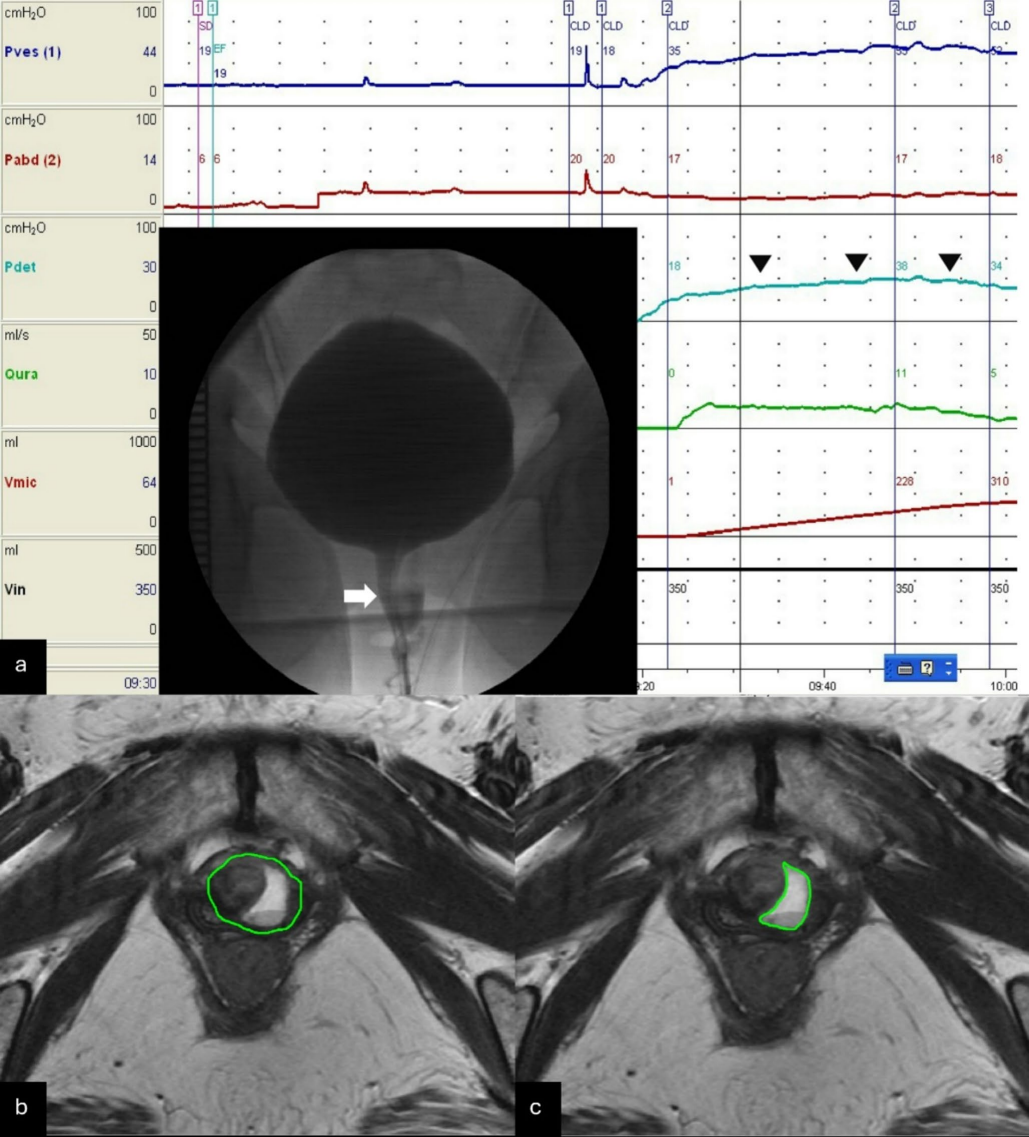
VUDS and MRI of the urethral diverticulum. (**a**) Voiding videocystometrogram displaying the urethral diverticulum with radiographic evidence of obstruction at the mid-urethra (arrow) during sustained detrusor contraction (arrowheads), indicating bladder outlet obstruction; (**b**) axial T2-weighted MRI showing the urethral sphincter complex and the urethral diverticulum; (**c**) axial T2-weighted MRI illustrating the urethral diverticulum

UD was categorized into proximal/middle UD (pmUD) and distal UD (dUD) (Online resource 1) based on the ostium’s location [8]. Bladder outlet obstruction (BOO) was defined as radiographic evidence of obstruction between the bladder neck and distal urethra, accompanied by a sustained detrusor contraction of any magnitude as shown by VUDS (Fig. 1a) [15]. High-pressure voiding status, defined as PdetQmax ≥ 20 cmH_2_O, —a detrusor pressure criterion from the Blaivas-Groutz nomogram—was used as an additional predictor of BOO [16].

The USCv was measured through T2-weighted MRI [8]. The urethra was outlined on consecutive slices from the bladder neck to the meatus, and its volume was calculated using segmentation tools in the image viewing software (SmartIris, Taiwan Electronic Data Processing Co., Taichung, ROC). Specifically, the diverticulum along with the urethral sphincter complex was outlined (Fig. 1b), and this volume was recorded (Volume A). Next, the diverticulum alone was outlined (Fig. 1c), and its volume was determined (Volume B). The USCv was then calculated by subtracting Volume B from Volume A.

Transvaginal diverticulectomy was performed in the lithotomy position under general anesthesia. An inverted U-shaped incision was made to reflect the anterior vaginal wall. The periurethral fascia was incised transversely and dissected laterally to facilitate complete mobilization of the diverticulum. The diverticular sac was carefully separated from the surrounding tissues. The ostium was identified, and complete excision of the urethral diverticulum was performed. The urethra was closed in a watertight fashion, followed by re-approximation of the periurethral fascia in a perpendicular orientation to the urethral closure line. A Martius flap was selectively utilized in cases with insufficient periurethral tissue or poor tissue quality. The vaginal wall was closed by re-approximating the anterior vaginal wall flap [7]. We routinely place a urethral Foley catheter along with a suprapubic cystostomy (except in cases of dUD) after diverticulectomy. The Foley catheter is removed four weeks later, based on the planned duration, and VUDS is performed immediately to assess urethral integrity. If periurethral contrast extravasation is identified, continued cystostomy drainage is recommended. If not, the cystostomy is clamped and removed three days later, provided the patient is voiding smoothly.

Patient characteristics were represented as n (%) or mean ± standard deviation. Basic statistical analyses of participant characteristics were performed using the Mann–Whitney *U* test for continuous variables and Fisher’s exact test for categorical variables. The Wilcoxon signed-rank test and linear regression analysis were utilized to compare categorical and continuous data before and after surgery. Receiver operating characteristic (ROC) curves assessed the relationship between USCv and treatment outcomes. All statistical analyses were conducted using SPSS Statistics version 29.0 (IBM Corp, Armonk, NY, USA), applying a two-sided test with a 5% significance level.

## Results

### Overall patient cohort

This study included 53 female patients with a mean age of 50.1 ± 15.16 years. The mean USCv and UDv were 9.1 ± 5.65 cm^3^ and 5.2 ± 7.00 cm^3^, respectively. Table 1 presents the clinical characteristics of the participants. Compared to the pmUD group (*n* = 43), the dUD group (*n* = 10) had a higher likelihood of presenting with a vaginal lump (37.2% vs. 80.0%, *p* = 0.031). Patients in the pmUD group reported a longer symptom duration than those in the dUD group (52.7 ± 91.15 months vs. 19.4 ± 24.56 months, *p* = 0.043). Additionally, the pmUD group had larger USCv (9.96 ± 5.91 cm^3^ vs. 5.23 ± 1.19 cm^3^, *p* < 0.001) and larger UDv (6.27 ± 7.40 cm^3^ vs. 0.84 ± 0.81 cm^3^, *p* < 0.001) compared to the dUD group. The mean operative time was significantly longer in the pmUD group than in the dUD group (230.14 ± 72.64 min vs. 127.0 ± 40.15 min, *p* < 0.001). Approximately 32.6% of patients in the pmUD group experienced UD recurrence on postoperative VUDS, while no patients in the dUD group had recurrence (*p* = 0.018). All cases of UD recurrence detected on postoperative VUDS were confirmed by MRI performed 3 to 6 months after surgery. All patients with recurrent UD had mild or no symptoms, and none of them underwent additional surgery. Although four patients (9.3%) in the pmUD group developed de novo stress urinary incontinence (SUI) after surgery, no patients in the dUD group experienced de novo SUI, and this difference was not statistically significant (*p* = 0.297).

**Table 1:** Participant characteristics, surgical parameters, and treatment outcomes

Characteristics	All patients (*n* = 53)	Proximal/Middle UD group (*n*= 43)	Distal UD group (*n* = 10)	*p*-value
Age (years) ± SD	50.1 ± 15.16	51.5 ± 13.36	43.8 ± 20.99	0.289
History of pelvic surgery, n (%)	17 (32.1)	16 (37.2)	1 (10.0)	0.140
Symptoms	-	-	-	-
IPSS (storage symptoms), n (%)	20 (37.7)	19 (44.2)	1 (10.0)	0.070
IPSS (voiding symptoms), n (%)	18 (34.0)	15 (34.9)	3 (30.0)	> 0.999
Urine leakage, n (%)	17 (32.1)	16 (37.2)	1 (10.0)	1.143
SUI, n (%)	12 (22.6)	12 (27.9)	0 (0.0)	0.093
Vaginal lump, n (%)	24 (45.3)	16 (37.2)	8 (80.0)	0.031^**^
Painful voiding, n (%)	21 (39.6)	18 (41.9)	3 (30.0)	0.722
Symptom duration (months) ± SD	46.3 ± 83.43	52.7 ± 91.15	19.4 ± 24.56	0.043^**^
History of rUTI, n (%)	20 (37.7)	19 (44.2)	1 (10.0)	0.070
Mean UDv (cm^3^) ± SD	5.2 ± 7.00	6.27 ± 7.40	0.84 ± 0.81	< 0.001^**^
Mean USCv (cm^3^) ± SD	9.1 ± 5.65	9.96 ± 5.91	5.23 ± 1.19	< 0.001^**^
Operative time (min) ± SD	210.7 ± 78.75	230.14 ± 72.64	127.0 ± 40.15	< 0.001^**^
Length of hospital stay (days) ± SD	4.5 ± 2.36	4.9 ± 2.44	2.9 ± 0.88	0.004^**^
Recurrence of UD (%)	14 (26.4)	14 (32.6)	0 (0.0)	0.018^**^
De novo SUI, n (%)	4 (7.5)	4 (9.3)	0 (0.0)	0.297
De novo UUI, n (%)	6 (11.3)	4 (9.3)	2 (20.0)	0.254
rUTI after surgery, n (%)	3 (5.7)	3 (7.0)	0 (0.0)	0.309

UD, urethra diverticulum; IPSS, International Prostate Symptom Score; rUTI, recurrent urinary tract infection; UDv, urethral diverticulum volume; USCv, urethral sphincter complex volume; SUI, stress urinary incontinence; UUI, urge urinary incontinence

**Table 2:** Relationship between clinical characteristics/treatment outcomes and UDv or USCv in the PmUD group

	UDv (cm^3^)	*p*-value	USCv (cm^3^)	*p*-value
A. Correlation between clinical characteristics and UDv or USCv				
Symptoms–IPSS (storage symptoms)		0.639		0.245
Yes	5.2 ± 6.80		10.2 ± 6.98	
No	7.1 ± 7.89		9.8 ± 5.05	
Symptoms–IPSS (voiding symptoms)		0.533		0.902
Yes	6.6 ± 5.72		10.3 ± 5.25	
No	6.1 ± 8.26		9.8 ± 6.32	
Symptoms: Urine leakage		0.214		0.174
Yes	5.5 ± 6.14		9.5 ± 4.52	
No	6.7 ± 8.14		10.2 ± 6.67	
Symptoms: SUI		0.442		0.137
Yes	4.8 ± 6.94		7.1 ± 4.26	
No	6.8 ± 7.61		11.1 ± 6.14	
Symptoms: Vaginal lump		0.528		0.296
Yes	6.1 ± 7.75		9.9 ± 5.10	
No	6.4 ± 7.34		10.0 ± 6.44	
Symptoms: Painful voiding		0.143		0.154
Yes	4.9 ± 5.75		8.9 ± 5.65	
No	7.2 ± 8.38		10.7 ± 6.08	
History of pelvic surgery		0.694		0.268
Yes	6.2 ± 7.07		10.36.61	
No	6.3 ± 7.73		9.5 ± 4.65	
History of rUTI		0.976		0.560
Yes	6.2 ± 6.92		9.1 ± 5.90	
No	6.3 ± 7.91		10.6 ± 5.96	
Preoperative BOO		0.288		0.594
Yes	5.4 ± 5.40		9.9 ± 4.68	
No	9.9 ± 11.55		11.3 ± 6.95	
Preoperative PdetQmax > 20 cmH_2_O		0.024^**^		0.011^**^
Yes	7.6 ± 7.97		11.2 ± 5.69	
No	1.9 ± 1.67		6.3 ± 1.50	
B. Correlation between treatment outcomes and UDv or USCv				
Recurrence of UD		0.572		0.341
Yes	6.2 ± 6.94		10.0 ± 5.53	
No	6.8 ± 8.26		11.0 ± 7.79	
De novo SUI		0.02^**^		0.089
Yes	11.5 ± 14.3		9.8 ± 5.8	
No	5.9 ± 6.4		14.7 ± 9.2	
De novo UUI		0.581		0.294
Yes	9.6 ± 10.91		13.8 ± 6.80	
No	6.1 ± 7.19		9.9 ± 6.15	
rUTI after surgery		0.70		0.348
Yes	5.7 ± 3.08		9.0 ± 2.42	
No	6.6 ± 7.89		10.5 ± 6.50	

UDv, urethral diverticulum volume; USCv, urethral sphincter complex volume; pmUD, proximal/middle urethra diverticulum; IPSS, International Prostate Symptom Score; rUTI, recurrent urinary tract infection; BOO, bladder outlet obstruction; SUI, stress urinary incontinence; UUI, urge urinary incontinence

### The PmUD group

Among the 43 female patients with pmUD, four cases (9.3%) had simple UD, 17 (39.5%) had U-shaped UD, and 22 (51.2%) had circumferential UD. Additionally, 24 were diagnosed with BOO, and 25 had high-pressure voiding status (Table 1). To further investigate the role of USCv, we analyzed the correlation between USCv, UDv, clinical manifestations, urodynamic characteristics, and treatment outcomes in the pmUD group. Table 2A summarizes the relationship between clinical characteristics and UDv/USCv in this group. There was no correlation between UDv and USCv with symptoms, history of pelvic surgery, recurrent urinary tract infection (rUTI), or the presence of BOO. However, patients with preoperative high-pressure voiding status exhibited larger UDv (7.6 ± 7.97 cm^3^ vs. 1.9 ± 1.67 cm^3^, *p* = 0.024) and USCv (11.2 ± 5.69 cm^3^ vs. 6.3 ± 1.50 cm^3^, *p* = 0.011).

**Fig. 2:**
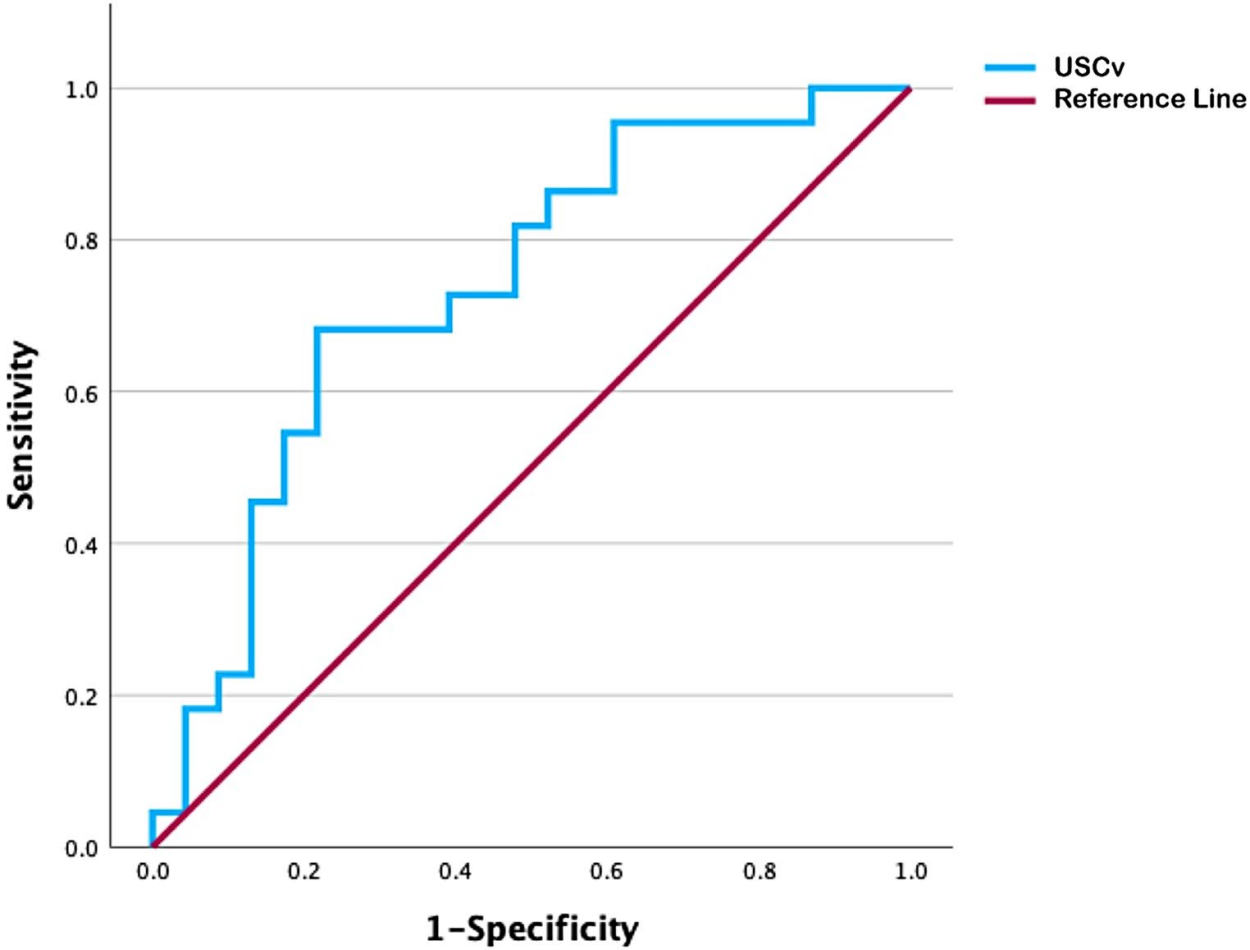
Receiver operating characteristic curve illustrating the sensitivity and specificity of urethral sphincter complex volume (USCv) in predicting unresolved high-pressure voiding status post-surgery in pmUD patients

After diverticulectomy, there were decreases in PdetQmax (35.1 ± 17.27 cmH_2_O vs. 28.7 ± 12.90 cmH_2_O, *p* = 0.05), increases in the free maximum flow rate (17.2 ± 9.40 ml/s vs. 21.1 ± 10.10 ml/s, *p* = 0.008), and decreases in postvoid residual (PVR) (29.1 ± 35.60 ml vs. 3.1 ± 7.24, *p* = 0.001) (Online Resource 2). Fourteen cases (32.6%) recurred. None (0/4) of the simple UDs recurred, but 11.8% (2/17) of the U-shaped and 54.5% (12/22) of the circumferential UD did. Twelve patients with preoperative BOO and seven with preoperative high-pressure voiding status did not improve after surgery. Linear regression analysis indicated that changes in PdetQmax, free maximum flow rate, and PVR were not associated with UDv or USCv (Online Resource 3). Regarding UD recurrence (14/43, 32.6%), unresolved BOO (12/24, 50.0%), and unresolved high-pressure voiding status (7/25, 28.0%), no correlation was found between these conditions and UDv or USCv (Table 2B). However, the ROC curve identified a USCv cutoff of ≥ 8.2 cm^3^, suggesting that high-pressure voiding status in patients with larger USCv was less likely to resolve (≤ 20 cmH_2_O) after surgery (sensitivity, 0.682; specificity, 0.800) (Fig. 2). The area under the curve was 0.755 (*p* = 0.023). Additionally, UD recurrence was more common in patients with USCv ≥ 8.2 cm³ (8/19, 42.1%) compared to those with USCv < 8.2 cm³ (6/24, 25%), though this difference was not statistically significant. Finally, four patients (9.3%) developed de novo SUI after surgery, and those with de novo SUI were associated with larger UDv (11.5 ± 14.3 cm^3^ vs. 5.9 ± 6.4 cm^3^, *p* = 0.02) but not USCv (9.8 ± 5.8 cm^3^ vs. 14.7 ± 9.2 cm^3^, *p* = 0.089). Additionally, four (9.3%) and three (7.0%) patients experienced de novo urge urinary incontinence (UUI) and persistent rUTI after surgery. However, UDv and USCv did not correlate with de novo postoperative UUI and rUTI (Table 2B).

## Discussion

Functional urethral obstruction caused by HTNRS may contribute to the development of UD proximal to the external urethral sphincter [8]. Therefore, we categorized UD into pmUD and dUD to evaluate the differences in clinical characteristics and treatment outcomes between the two groups. In this study, 80% of female patients with dUD reported a vaginal lump, which allowed for earlier diagnosis and a shorter duration of symptoms. Additionally, women with pmUD were more likely to have larger USCv and UDv compared to those with dUD. Thus, diverticulectomy for pmUD patients required more time and had a higher risk of UD recurrence. The increased USCv and UDv in women with pmUD suggest that HTNRS may play a role in the formation of pmUD and complicate treatment. To our knowledge, no prior literature has examined the impact of UD location on clinical characteristics and treatment outcomes.

In this study, we evaluated urethral pressure and obstructive voiding using PdetQmax and the fluoroscopic appearance of the urethra. We focused on detrusor pressure alone instead of combining it with flow data, as women are more likely to use abdominal straining, which can significantly impact flow rates [17]. Our findings indicated that over 50% of patients with pmUD were diagnosed with BOO and/or high-pressure voiding status. While the presence of BOO did not correlate with UDv or USCv, high-pressure voiding status was associated with larger UDv and USCv. BOO diagnosis relied on the fluoroscopic identification of a narrowed area in the urethra along with proximal dilatation. This diagnosis may introduce interpreter bias, especially in patients with UD, leading to distorted urethras and unclear urethra delineation. PdetQmax may serve as a better predictor of urethral pressure in these patients. Theoretically, obstructive voiding could stem from the obstructive or mass effects of UD on the urethra, explaining the association between high-pressure voiding status and larger UDv. Additionally, the correlation between high-pressure voiding status and larger USCv suggests that HTNRS creates high-pressure in the proximal urethra during voiding and may contribute to pmUD formation.

In our study, diverticulectomy led to significant reductions in PdetQmax; however, these changes were not correlated with UDv or USCv. In addition, 50% of patients with BOO and 28% of those with high-pressure voiding status did not experience resolution after the procedure. Furthermore, neither UDv nor USCv, when analyzed as continuous variables, could predict postoperative unresolved BOO or high-pressure voiding status. However, when USCv was dichotomized using a cutoff value of 8.2 cm^3^, a larger USCv (≥ 8.2 cm^3^) was found to predict unresolved high-pressure voiding status post-diverticulectomy. This suggests that obstructive voiding may not be solely dependent on the obstructive effects of UD on the urethra, and diverticulectomy may not fully resolve all cases of obstructive voiding related to UD. A USCv of ≥ 8.2 cm^3^ indicates that diverticulectomy alone may be insufficient for addressing obstructive voiding due to HTNRS-related UD. Treatments such as sacral neuromodulation [18] or intraurethral botulinum toxin [19] may be considered after UD excision to mitigate the risk of recurrence. We can inform women with UD and USCv ≥ 8.2 cm^3^ about the potential complications of unresolved high-pressure voiding status and the possibility of requiring additional treatment.

In this study, fourteen cases (32.6%) in the pmUD group experienced recurrence of UD on postoperative VUDS. None (0/4) of the simple UDs recurred, but 11.8% (2/17) of the U-shaped and 54.5% (12/22) of the circumferential UD did. Reported recurrence rates vary widely in the literature, with Ingber et al. [20] noting a rate of 10.7%, while Han et al. [8] report rates as high as 60% in cases of circumferential UD. Han et al. reported a recurrence rate of 23.3% (7 out of 30 cases) among UD patients diagnosed via MRI or transvaginal ultrasonography 3 months after diverticulectomy [21]. None of the simple UD cases recurred, whereas 33% of U-shaped and 60% of circumferential UD cases did. Therefore, the recurrence rate of UD after diverticulectomy in our study is comparable to that reported in the literature, particularly when UD type is taken into account.

In the present study, neither UDv nor USCv could predict UD recurrence. Furthermore, preoperative factors such as the location, multiplicity, and size of the UD did not influence surgical outcomes. Recurrence has traditionally been associated with surgical technique, diverticulum characteristics (including complexity, position, or burden), and patient factors like poor tissue quality or high body mass index [8]. However, HTNRS should also be considered. If HTNRS causes increased urethral pressures that contribute to pmUD and is not addressed, the likelihood of recurrence over time is higher. In our study, UD recurrence occurred exclusively in patients with pmUD, with none observed in those with dUD. Additionally, recurrence was more common in patients with USCv ≥ 8.2 cm³ (8/19, 42.1%) compared to those with USCv < 8.2 cm³ (6/24, 25%), although this difference was not statistically significant. El-Nashar et al. discovered that baseline dysuria, a frequent symptom of HTNRS, increased the likelihood of recurrence after diverticulectomy [22]. Two studies have shown that recurrence is more probable in proximal UD [20, 23]. Therefore, unrecognized and untreated HTNRS may play a role in the recurrence of UD following diverticulectomy.

In our study, approximately 10% of patients with pmUD developed de novo SUI, which was associated with UDv. Other common adverse events included de novo UUI (*n* = 4, 9.3%) and rUTI (*n* = 3, 7.0%). De novo SUI has been reported to occur in 1.7–33% of patients undergoing transvaginal urethral diverticulectomy [24]. Contributing factors may include extensive dissection in large diverticula and their proximity to the urethra, which can compromise the support structures of the urethra and bladder neck. Additionally, denervation during surgery or damage to the urethral muscles and bladder neck from inflammation or the diverticulum itself may result in postoperative SUI [25]. Several studies have supported these theories [25–27], indicating an increased risk of de novo SUI in large, proximally located, or circumferential diverticula. Similarly, our findings showed that de novo SUI correlated with UDv, whereas diverticulectomy for dUD did not lead to de novo SUI. Barratt et al. conducted a retrospective review of 122 patients who underwent surgical excision of UD and found that 1.8% (*n* = 2) experienced de novo detrusor overactivity incontinence [28]. The incidence of de novo UUI in our study appeared to be significantly higher than that reported in Barratt’s series. However, we defined UUI based on presenting symptoms rather than urodynamic findings. Among the four patients with de novo UUI in our study, only one (2.3%) showed urodynamic detrusor overactivity. Additionally, three patients (7%) experienced unresolved rUTI after diverticulectomy. Most cases of postoperative rUTI were persistent from before the urethral diverticulectomy rather than being new occurrences, with rates of persistence reaching up to 23% [25, 26].

There are several inherent limitations to this retrospective review of a rare condition. The study includes a small sample size from a single institution, and we lacked complete data on postoperative symptom scores and long-term outcomes. Residual tissue inflammation one month after diverticulectomy may have contributed to some of the residual obstruction observed on VUDS. Despite these limitations, few studies have investigated the alternative etiology of UD, HTRNS, and its effects on clinical manifestations, urodynamic characteristics, and treatment outcomes in women with UD. Additionally, we established a cutoff value of 8.2 cm³ for USCv to predict unresolved high-pressure voiding status after diverticulectomy, which may aid in preoperative counseling about potential additional interventions, either during or after the procedure.

## Electronic supplementary material

Below is the link to the electronic supplementary material.

Supplementary Material 1

Supplementary Material 2

Supplementary Material 3

## Data Availability

No datasets were generated or analysed during the current study.
