# Supplementary material for: Predictive value of urethral sphincter complex volume for persistent high voiding pressure in female urethral diverticulum patients post-diverticulectomy
Source: World J Urol. 2025 Jun 5;43(1):358. doi: 10.1007/s00345-025-05719-w (PMC12141153; doi:10.1007/s00345-025-05719-w)

**Online Resource 1. Magnetic resonance imaging scans of urethral diverticulum (UD): axial (a1) and sagittal (a2) view of proximal/middle UD; axial (b1) and sagittal (b2) view of distal UD.**


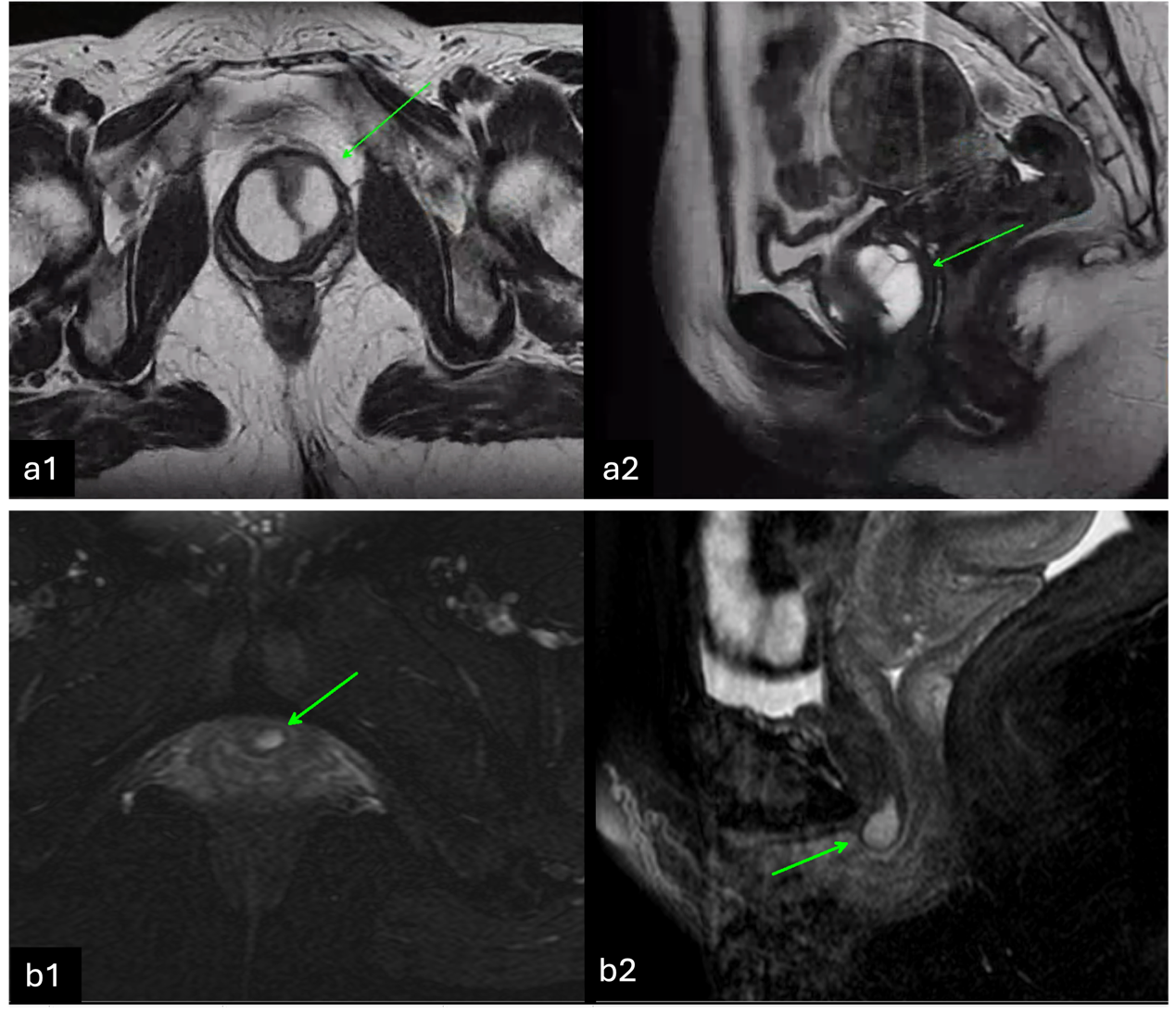

Supplement: Supplementary file 1 — Supplementary Material 1 [file 345_2025_5719_MOESM1_ESM.docx]
