# Supplementary material for: Predictive value of urethral sphincter complex volume for persistent high voiding pressure in female urethral diverticulum patients post-diverticulectomy
Source: World J Urol. 2025 Jun 5;43(1):358. doi: 10.1007/s00345-025-05719-w (PMC12141153; doi:10.1007/s00345-025-05719-w)
Supplement: Supplementary file 2 — Supplementary Material 2 [file 345_2025_5719_MOESM2_ESM.docx]

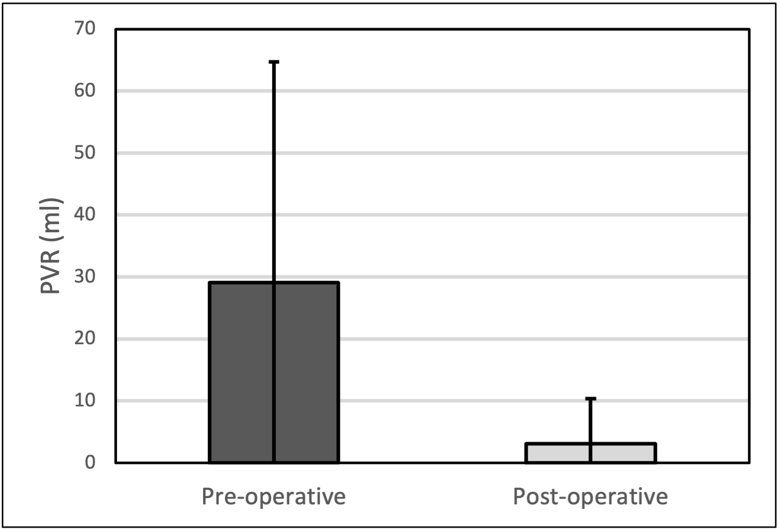

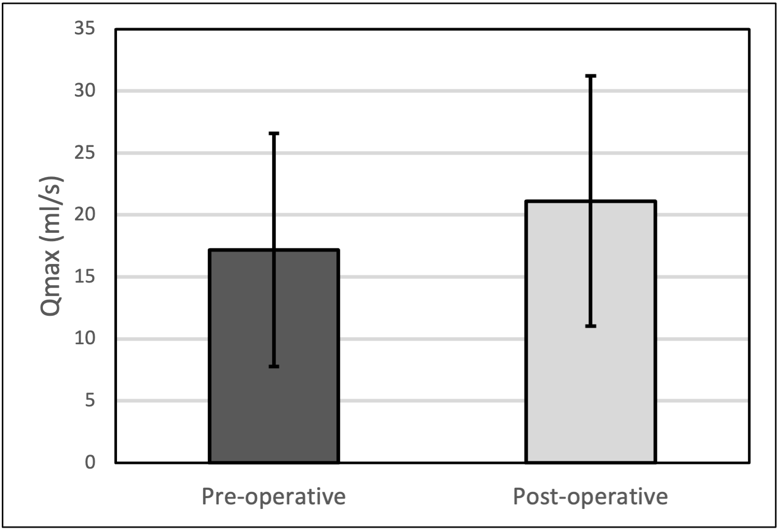

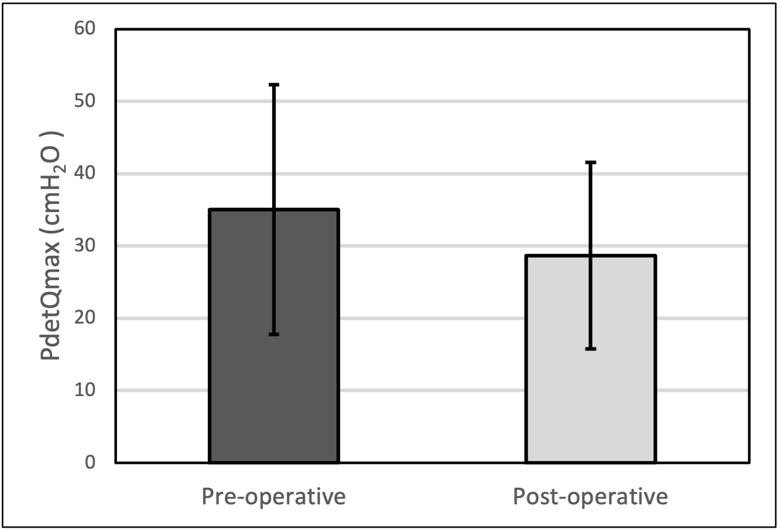
**Online Resource 2. Changes in (A) PdetQmax, (B) free Qmax, and (C) postvoid residual (PVR) pre- and post-surgery in pmUD patients**

*p*=0.001

*p*=0.05

*p*=0.008

C

A

B
