# Supplementary material for: Predictive value of urethral sphincter complex volume for persistent high voiding pressure in female urethral diverticulum patients post-diverticulectomy
Source: World J Urol. 2025 Jun 5;43(1):358. doi: 10.1007/s00345-025-05719-w (PMC12141153; doi:10.1007/s00345-025-05719-w)
Supplement: Supplementary file 3 — Supplementary Material 3 [file 345_2025_5719_MOESM3_ESM.docx]

**Online Resource 3. Linear regression analysis of USCv and UDv, along with changes in PdetQmax, free Qmax, and postvoid residual (PVR) before and after surgery in the pmUD group**

| **PdetQmax changes** | | | |
| --- | --- | --- | --- |
|  | **Partial regression coefficient (β)** | **Standard error (SE)** | ***p*-value** |
| **USCv** | –0.179 | 0.531 | 0.740 |
| **UDv** | –0.152 | 0.371 | 0.687 |
| **Qmax changes** | | | |
|  | **Partial regression coefficient (β)** | **Standard error (SE)** | ***p*-value** |
| **USCv** | 0.082 | 0.194 | 0.677 |
| **UDv** | –0.132 | 0.135 | 0.337 |
| **PVR changes** | | | |
|  | **Partial regression coefficient (β)** | **Standard error (SE)** | ***p*-value** |
| **USCv** | 0.142 | 0.950 | 0.882 |
| **UDv** | 1.095 | 1.302 | 0.408 |
